# Are large clinical trials in orthopaedic trauma justified?

**DOI:** 10.1186/s12891-018-2029-3

**Published:** 2018-04-20

**Authors:** Sheila Sprague, Paul Tornetta, Gerard P. Slobogean, Nathan N. O’Hara, Paula McKay, Brad Petrisor, Kyle J. Jeray, Emil H. Schemitsch, David Sanders, Mohit Bhandari, on behalf of the FLOW Investigators, Mohit Bhandari, Gordon H. Guyatt, Kyle J. Jeray, Stephen Walter, Brad Petrisor, Emil H. Schemitsch, Paul Tornetta, Jeff Anglen, Michael Bosse, Susan Liew, Parag Sancheti, Sheila Sprague, Paula McKay, Kim Madden, Kerry Tai, Diane Heels-Ansdell, Lisa Buckingham, Aravin Duraikannan, Stephanie L. Tanner, Rebecca G. Snider, Douglas Altman, Rajiv Gandhi, Markus Bischoff, Gregory J. Della Rocca, Bill Ristevski, Krishan Rajaratnam, Dale Williams, Matthew Denkers, Drew Bednar, John Sadler, Desmond Kwok, Brian Drew, Ivan Wong, Jeremy A. Hall, Michael D. McKee, Henry Ahn, Daniel Whelan, James Waddell, Timothy Daniels, Earl Bogoch, Aaron Nauth, Milena R. Vicente, Jennifer T. Hidy, David Sanders, Abdel-Rahman Lawendy, Kevin Gurr, Timothy Carey, Chris Bailey, Mark Macleod, Debra Bartley, Christina Tieszer, Chad Coles, Ross Leighton, C. Glen Richardson, Michael Biddulph, Michael Gross, Michael Dunbar, J. David Amirault, David Alexander, Catherine Coady, Mark Glazebrook, David Johnston, William Oxner, J. Andrew Trenholm, Gerald Reardon, Kelly Trask, Shelley MacDonald, Steven Papp, Wade Gofton, Allan Liew, Stephen Kingwell, Joseph O’Neill, Garth Johnson, Eugene Wai, Julia Foxall, Henry M. Broekhuyse, Peter J. O’Brien, Piotr A. Blachut, Kelly A. Lefaivre, Raman Johal, Stéphane Leduc, G. Yves Laflamme, Pierre Beaumont, Michel Malo, Benoit Benoit, Dominique Rouleau, Pierre Ranger, Julie Fournier, Karine Tardif, Rudy Reindl, Greg Berry, Edward Harvey, William Fisher, Mark Burman, Paul Martineau, Eric Lenczner, Robert Marien, Robert Turcotte, Michael Tanzer, Max Talbot, Peter Jarzem, Mike Weber, Fiona Houghton, Robert McCormack, Kelly Apostle, Dory Boyer, Farhad Moola, Bertrand Perey, Trevor Stone, Darius Viskontas, H. Michael Lemke, Mauri Zomar, Karyn Moon, Raely Moon, Hans Kreder, Richard Jenkinson, David Stephen, Markku Nousiainen, Terry Axelrod, Veronica Wadey, Michael Ford, Joel Finkelstein, Richard Holtby, Robin Richards, Sebastian Rodriguez-Elizalde, Diane Nam, Albert Yee, Patrick Henry, John Murnaghan, Harsha Malempati, Julian Sernik, Tim Dwyer, Katrine Milner, Monica Kunz, Melanie MacNevin, Wesley Ghent, Fathima Adamsahib, Ria De Gorter, Michelle Arakgi, Ted V. Tufescu, Brad Pilkey, Chris Graham, Laurie Barron, Allan Hammond, Nigar Sultana, Ryan T. Bicknell, David Pichora, Aaron Campbell, Fiona Howells, Annie Deshaies, Frédéric Balg, François Cabana, Rejean Dumais, Jean-François Joncas, Marc-André Magalhaes-Grave, Nicolas Patenaude, Bernard LaRue, Stéphane Ricard, Chantal Théorêt, François Vézina, Amy Svotelis, Jennifer Downey, Stéphane Pelet, Jean Lamontagne, Luc Bédard, Alexandre Denault, Pierre Lavallée, Luc Petitclerc, Bernard Laliberté, Martin Bédard, Marie-Eve Roger, Luc Lemire, Hélène Côté, Linda Lépine, Pascale Lévesque-Bernier, J. Scott Broderick, David R. Goetz, Thomas M. Schaller, Scott E. Porter, Michael L. Beckish, John D. Adams, Benjamin B. Barden, Grant W. Bennett, David M. Conner, Aaron T. Creek, Melissa M. Earles, Stephen H. Finley, Jonathan L. Foret, Garland K. Gudger, Richard W. Gurich, Austin D. Hill, S. Matthew Hollenbeck, Lyle T. Jackson, Benjamin S. Koch, Kevin K. Kruse, Wesley G. Lackey, Justin W. Langan, Julia Lee, Lauren C. Leffler, Michael J. Maughon, S. Brennan McClure, Timothy J. Miller, R. Lee Murphy, Lawrence K. O’Malley, Dustin M. Price, Lorra M. Sharp, J. Adam Smitherman, John A. Tanksley, Erick G. Torres, Dylan J. Watson, Scott T. Watson, Shea A. Bielby, Lauren A. Nastoff, Robert J. Teasdall, Joseph Hsu, Katherine M. Bedigrew, Tod Gerlinger, Dave Brown, Joseph Alderete, Kevin Kirk, Mickey Cho, Anthony Johnson, Raymond Topp, Damian Rispoli, James Ficke, Eric Ritchie, Anthony Beardmore, Siraj Sayeed, Michael Charlton, Kristen Walick, Dmitry Tuder, Greg Maytok, Travis Burns, Donald Gajewski, Warren Kactmas, Ramnov Andreson, Patrick Osborn, Michael Connally, Donna Lopez, Mary Fan, Dennis Mann, Andrea Garza, Rina L. Harman, Steven Olson, Robert Zura, Rachel Reilly, Prerana Patel, Claude T. Moorman, Fraser Leversedge, Chard Harbour, Brian Brigman, David Ruch, Nikoletta Leontaritis, Michael Bolognesi, Shalini Ramasunder, Alison Toth, Allen Diane, Grant Garrigues, Dean Taylor, Richard C. Mather, Kristoff Reid, Robert Lark, Samuel Adams, Maria Manson, Utku Kandemir, Saam Morshed, Murat Pekmezci, Richard Coughlin, Trigg McClellan, Meir Marmor, Eric Meinberg, Tigist Belaye, Jonathan Kwong, Clifford B. Jones, James R. Ringler, Terrence J. Endres, David J. Bielema, Michael R. Jabara, Samuel G. Agnew, Debra L. Sietsema, Jane E. Walker, Brett D. Crist, Yvonne M. Murtha, David A. Volgas, James P. Stannard, Linda K. Anderson, Kelly M. Sullivan, Lori Kramer Clark, Kathleen Markley, Stacee Clawson, Andrew Schmidt, Patrick Yoon, Thomas Varecka, Matthew Karam, Jerald R. Westberg, Lisa K. Cannada, Jason Stoneback, Kevin Kuhn, Erik Nott, Leslie Dillender, Karl Shively, Brian Mullis, Janos Ertl, Ripley Worman, Jeffrey Anglen, Valda Frizzell, Molly Moore, Michael J. Prayson, David Nelles, Jason Vourazeris, Matthew Ross, Richard T. Laughlin, Joseph Cox, Roman Trimba, Joy M. Bradford-Johnson, Andrew J. Marcantonio, Michael Kain, Richard Wilk, Mark Lemos, Joshua Baumfeld, John Tilzey, Brian Jolley, John Garfi, Ivan Tarkin, Andrew Evans, Peter Siska, Lisa Blackrick, Dana J. Farrell, Emily Keener, Jason Lowe, William Min, Jeffrey Leary, Rena Stewart, David Volgas, Leslie Barnes, Nurit Shadmi, Matthew Robinson, Taylor Vlack, Kathryn Hornbuckle, Melanese Leonard, Nikia Hawkins Malone, Tanya Nix, Jessica Goldstein, David Zamorano, Martin Tynan, Samuel Bederman, Nitin Bhatia, Arthur Kreitenberg, Bang Hoang, Deeba Pourmand, Deanna Lawson, Anthony Rhorer, Brian Miller, Gilbert Ortega, Lori Wood, Veronica Place, Harvinder Bedi, Ashley Carr, Andrew Chia, Hamish Curry, Steve Csongvay, Craig Donohue, Stephen Doig, Elton Edwards, Eugene Ek, Max Esser, Greg Etherington, Richard Freeman, Andrew Gong, Doug Li, Matthan Mammen, Russell Miller, Ash Moaveni, Mathias Russ, Lu Ton, Tom Treseder, Otis Wang, Zoe Murdoch, Claire Sage, Adam Dowrick, John Clarke-Jenssen, Frede Frihagen, Lars Nordsletten, Tor Nicolaysen, Hilde Apold, Petter Iversen, Are Stodle, Mette Andersen, Vera Halvorsen, Geir Hjorthaug, Anders Lippert, Ida Sletten, Ellen Langslet, Marius Molund, Asgeir Amundsen, Oliver Muller, Cathrine Aga, Torben Ianssen, Gunnar Flugsrud, Jonas Rydinge, Kim Hemlock, Jan Egil Brattgjerd, John Magne Hoseth, Bernhard Flatoy, Havard Furunes, Peder Bogsti, Guri Ekås, Gilbert Moatshe, Ali Al-Ashtari, Tore Fjalestad, Fredrik Nilsen, Morten Smedsrud, Anne Christine Brekke, Elise Berg Vesterhus, Sissel Knuts, Steve Rocha, Chetan Puram, Atul Patil, Neelam Jhangiani, Anil K. Rai, Kamal Narayan Rai, Vivek V. Jabade, Deepali Nassikars, Narayan J. Karne, Chetan Metha, A. Navaladi Shankar, R. Saravana, Ajay Gupta, Neeraj Jain, Mahesh Bhatia, Vinod Arora, Vivek Tyagi, Anoop Dubey, Vinit Yadav, Rani Rai, Kiran M. Doshi, Arjun Patil

**Affiliations:** 1 Division of Orthopaedic Surgery, Department of Surgery, McMaster University, Hamilton, ON Canada; 2 Department of Health Research Methods, Evidence, and Impact, McMaster University, Hamilton, ON Canada; 3 Department of Orthopaedic Surgery, Boston University School of Medicine, Boston, MA USA; 4 Department of Orthopaedics, University of Maryland School of Medicine, R Adams Cowley Shock Trauma Center, Baltimore, MD USA; 5 Department of Orthopaedic Surgery, Greenville Health System, Greenville, SC USA; 6 Department of Surgery, University of Western Ontario, London, ON Canada

**Keywords:** Large trials, Orthopaedic trial, Sample size, FLOW trial

## Abstract

**Background:**

The objective of this analysis is to evaluate the necessity of large clinical trials using FLOW trial data.

**Methods:**

The FLOW pilot study and definitive trial were factorial trials evaluating the effect of different irrigation solutions and pressures on re-operation. To explore treatment effects over time, we analyzed data from the pilot and definitive trial in increments of 250 patients until the final sample size of 2447 patients was reached. At each increment we calculated the relative risk (RR) and associated 95% confidence interval (CI) for the treatment effect, and compared the results that would have been reported at the smaller enrolments with those seen in the final, adequately powered study.

**Results:**

The pilot study analysis of 89 patients and initial incremental enrolments in the FLOW definitive trial favored low pressure compared to high pressure (RR: 1.50, 95% CI: 0.75–3.04; RR: 1.39, 95% CI: 0.60–3.23, respectively), which is in contradiction to the final enrolment, which found no difference between high and low pressure (RR: 1.04, 95% CI: 0.81–1.33). In the soap versus saline comparison, the FLOW pilot study suggested that re-operation rate was similar in both the soap and saline groups (RR: 0.98, 95% CI: 0.50–1.92), whereas the FLOW definitive trial found that the re-operation rate was higher in the soap treatment arm (RR: 1.28, 95% CI: 1.04–1.57).

**Conclusions:**

Our findings suggest that studies with smaller sample sizes would have led to erroneous conclusions in the management of open fracture wounds.

**Trial registration:**

NCT01069315 (FLOW Pilot Study) Date of Registration: February 17, 2010, NCT00788398 (FLOW Definitive Trial) Date of Registration: November 10, 2008.

## Background

Large definitive clinical trials in orthopaedic trauma are expensive, challenging, and time consuming to conduct. In times of limited research funding, their value may be called into question as it costs several million dollars to answer one or two clinical questions and results may not be translated into practice for five to eight years following initiation of the trial [1]. However, it is equally important to consider that smaller trials may be inadequately powered to answer clinical questions or have fragility in their results, potentially leading to an over or underestimation of the true treatment effect. Small studies are subject to Beta error, which is to say that no difference is found even though one may exist. While this is considered a less egregious error than a false finding of a difference in treatments, if one treatment is in fact better than another and this is not found due to small sample size, an opportunity to improve patient care is missed.

Previous research has demonstrated that it is not uncommon for the results of highly cited clinical studies published in high impact medical journals to be contradicted by the findings of subsequent studies [2]. Randomized controlled trials (RCTs) are not immune from having their findings contradicted by subsequent studies, with small RCTs being at higher risk than larger ones of having their findings contradicted by subsequent trials [2, 3]. The objective of this study is to evaluate the value of one large clinical trial in orthopaedic trauma using data from the FLOW trial.

## Methods

This investigation was part of the multi-centre FLOW (Fluid Lavage in Open Fracture Wounds) initiative. As a means of proving feasibility and testing the FLOW protocol, we completed the FLOW pilot study. The FLOW pilot study was a 2 × 2 factorial design which evaluated irrigation solution (soap vs. normal saline) and irrigation pressure (low vs. high) in patients with open fracture wounds. The FLOW pilot study included 89 patients at nine clinical sites in Canada and the United States. The results of the FLOW pilot study (ClinicalTrials.gov number, NCT01069315) informed the development of the FLOW definitive trial (ClinicalTrials.gov number, NCT00788398), which was a multi-center, blinded, randomized controlled trial, using a 2 × 3 factorial design which evaluated irrigation solution (soap vs. normal saline) and irrigation pressure (very low vs. low vs. high) in patients with open fracture wounds [4]. The FLOW definitive trial included 2447 patients across 41 clinical sites in the United States, Canada, Australia, Norway, and India [5]. The primary outcome for the FLOW pilot and definitive studies was re-operation within 12 months of fracture to treat infection or promote wound or fracture healing. Research Ethics Board (REB) Approval for the FLOW pilot and definitive trial was obtained at the coordinating centre (McMaster University) (REB: 05–299 and 08–268) and at each clinical site. All procedures followed were in accordance with ethical standards of the ethics boards.

To evaluate the value of a trial of this size, we analyzed the data from the pilot study and then the definitive trial in increments of 250 patients until the final sample size was reached. Increments of 250 were selected for ease of reporting and to reflect what the findings would have been for a trial of each incremental size. At each increment, we calculated the relative risk (RR) and associated 95% confidence interval (CI) for the treatment effect. We then compared the results that would have been reported at the smaller enrolments with those seen in the final, adequately powered study. All analyses were performed using JMP Version 12.0 (Cary, NC).

## Results

**Fig. 1:**
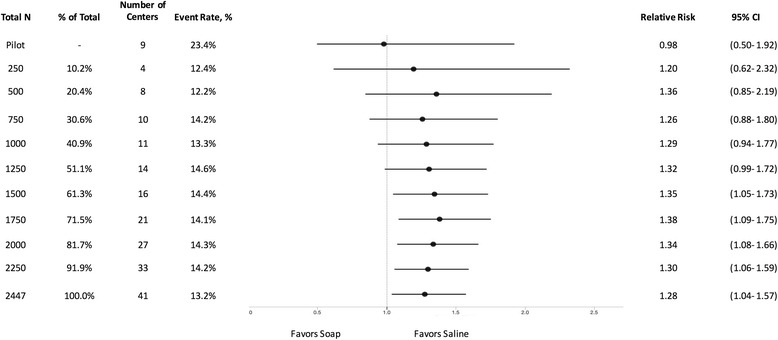
Effect of soap vs. saline on FLOW trial patients at different sample sizes

In the soap versus saline comparison, data from the FLOW pilot study suggested that a small benefit to irrigation with soap may exist (RR: 0.98, 95% CI: 0.50–1.92, *p* = 0.82). However, the wide confidence intervals on each side of the relative risk indicated similar likelihood that soap or saline could be more efficacious. These preliminary results based on a very small sample size were sharply contrasted by the larger definitive FLOW trial which found that the risk of re-operation was higher in the soap treatment arm (RR: 1.28, 95% CI: 1.04–1.57, *p* = 0.02) (Fig. 1). The results achieved statistical significance (with the lower confidence interval for the relative risk being greater than one) when 1500 patients were enrolled into the trial (*p* = 0.02). As expected, the confidence intervals narrowed as the final sample size of 2447 was reached.

**Fig. 2:**
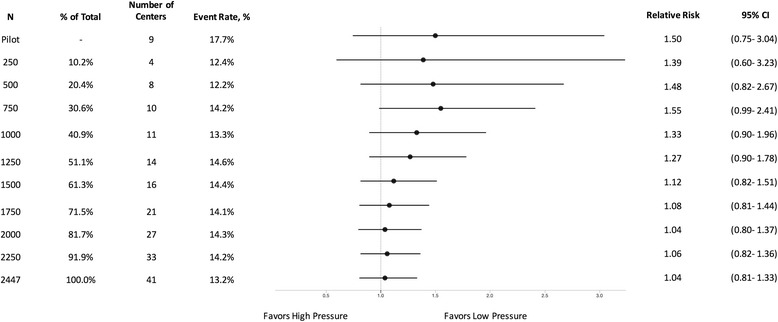
Effect of high vs. low pressure on FLOW trial patients at different sample sizes

**Fig. 3:**
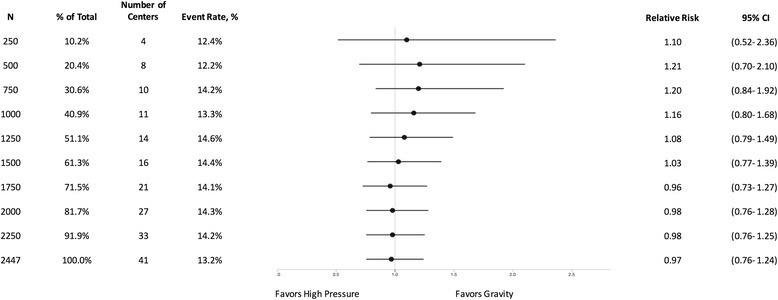
Effect of high vs. very low pressure on FLOW trial patients at different sample sizes

**Fig. 4:**
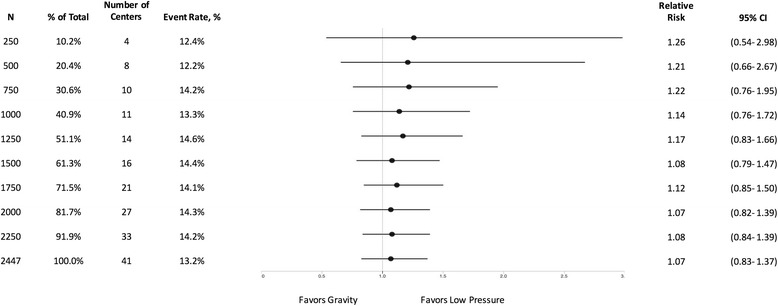
Effect of low vs. very low pressure on FLOW trial patients at different sample sizes

The results of all three pressure comparisons demonstrated similar patterns in which the initial increments of patients favored one pressure over another but the final enrolment of 2447 patients found no difference between irrigation pressure treatment groups (Figs. 2, 3 and 4). For example, the pilot study analysis of 89 patients favored low pressure compared to high pressure irrigation (RR: 1.50, 95% CI: 0.75–3.04 (*p* = 0.14) (Fig. 2). The initial incremental enrolments in the definitive trial also favored low pressure compared to high pressure (e.g. *n* = 250, RR: 1.39, 95% CI: 0.60–3.23, *p* = 0.48). When 750 patients were enrolled into the trial, the difference approached significance with *p* = 0.06 (RR = 1.55, 95% CI: 0.99–2.41). This is in contradistinction to the final enrolment of 2447 that found no difference between high and low pressure (RR: 1.04, 95% CI: 0.81–1.33, *p* = 0.82).

Similarly, the initial increment of 250 patients favored very low pressure over high pressure (RR: 1.10, 95% CI: 0.52–2.36, *p* = 0.83) (Fig. 3), and low pressure over very low pressure (RR: 1.26, 95% CI: 0.54–2.98, *p* = 0.63) (Fig. 4). However, in both cases, the final enrolment demonstrated no differences between any of the irrigation pressures.

## Discussion

Our findings suggest that both the results of the FLOW pilot study and selecting a smaller sample size for the FLOW definitive trial would have led to erroneous conclusions in the management of open fracture wounds. With regard to the results of one of the major research questions, namely whether irrigation with soap reduced the re-operation rate, the point estimate changes from potentially favoring soap to demonstrating that irrigation with soap is a risk factor for re-operation in the final enrollment increments of the definitive trial. At lower enrolment increments, comparisons between different pressures initially favored low pressure over both high and very low pressure, and very low pressure over high pressure. However, no differences between any of the irrigation pressures were observed at the final enrolment of 2447 patients. Most notably, at 750 patients, the results came very close to demonstrating a significant difference favoring low pressure over high pressure despite the fact that no significant differences were observed at higher enrolment increments.

When interpreting these results, it is important to consider that we analyzed the data in sequential increments of 250 patients. Since the number of clinical sites and participating surgeons increased with increasing sample size, differences in performance variables between sites and surgeons may have contributed to the observed differences in outcomes. If we had repeated these analyses using randomly selected subsets of patients, the results may have been quite different. However, this approach would not have represented an accurate reflection of the number of centers, participating surgeons, or enrollment timelines typically associated with smaller RCTs.

The sample size for the pilot study (*n* = 89) was chosen to inform the design of a large definitive trial and demonstrate to large funding agencies that the selected methodology was feasible. We published the results of the FLOW pilot study to generate interest and promote the definitive trial among the orthopaedic trauma community. We did not publish the pilot study results with the goal of changing clinical practice. Had this been our intent, erroneous treatment recommendations would have been made. In contrast, the sample size for the FLOW definitive trial was based on a sample size calculation, where we carefully considered realistic a priori event rates and corresponding reductions in relative risks between the treatment groups based upon the previous literature and the opinion of multiple experts [4].

The FLOW study was a factorial study and the advantages of a factorial design over a standard parallel group design include the ability to efficiently investigate a minimum of two interventions and the reduction in the total number of patients needed to assess multiple interventions aimed at achieving the same outcome. If we had not used a factorial study, the sample size for an adequately powered study, using the assumptions described above, comparing soap versus saline would have been 1200 patients (600 patients per treatment arm). Similarly, a separate study comparing high versus low versus very low pressure would have required 2280 patients (760 per treatment arm). Consequently, answering the two research questions in two separate studies would have required 3480 patients, demonstrating the efficiency of a factorial design.

As demonstrated in Figs. 1, 2, 3 and 4, it is evident that the results of the FLOW study appear to stabilize before the final enrolment of 2447 patients. Specifically, in the soap versus saline comparison the results seem to stabilize around 1200 patients, which is consistent with the above sample size calculation. The results for the comparisons between the three irrigating pressures seem to stabilize following the enrolment of approximately 2000 patients. One may question whether we enrolled too many patients into the FLOW trial. The answer is “no” as this stabilization occurred because the overall FLOW study sample size was calculated based upon the three pairwise comparisons for the pressures, however, in the end, the hypothesized differences between pressures were not observed. Had the results of the FLOW trial shown our hypothesized differences, it is possible that this stabilization would not have occurred until closer to the final sample size. In order to explore treatment effects over time, we have analyzed the data from the FLOW trial in small increments. However, if investigators conduct repeated interim analyses during the course of a trial without adjusting the significance level, there is a very real risk of observing a significant result merely by chance [6]. To mitigate this risk, it is important that investigators follow established early stopping rules that require more stringent significance levels when conducting interim analyses.

There has been a recent focus on large RCTs among the orthopaedic community, as evident by the Orthopaedic Trauma Association’s decision to feature a symposium that only included the findings from large RCTs at their 2015 Annual Meeting. Different trial groups have been established (i.e. COTS, METRC, OTRC) with mandates of conducting large, multi-centre RCTs. Despite this shift, the orthopaedic literature continues to be dominated by studies that are underpowered to guide current evidence. While many readers understand that trials may be underpowered, few realize that truly erroneous conclusions may come from smaller studies, including a reversal of the initial findings. In a review of 47 highly cited research studies published in 3 high impact medical journals, Ioannidis found that of the 45 studies that claimed the intervention in question was effective, seven (16%) were contradicted by subsequent studies and seven others (16%) demonstrated effects that were stronger than those found in future studies [2]. Among the RCTs included in his review, those with contradicted or stronger effects were significantly smaller (*p* = 0.009) than those whose effects were either replicated by future studies or whose findings remained unchallenged by subsequent studies. Similar findings have been observed within the orthopaedic literature, in that trials with smaller sample sizes and lower event rates have been shown to observe larger treatment effects in their results [7]. Similar to our FLOW analysis, an analysis of data from the SPRINT trial [8] found a smaller sample size for the SPRINT trial would have led to misleading estimates of the relative risk of reoperation between reamed and unreamed nails in the management of closed tibial shaft fractures [3]. As demonstrated by the results of the review by Ioannidis, the SPRINT analysis, and the current analyses of the data from the FLOW trial, readers need to consider the adequacy of the sample size when reviewing the results of RCTs.

Specifically, readers should consider whether the sample size calculation was based upon realistic estimates of event rates and meaningful risk reductions. In addition, readers should consider whether the sample size calculation is based upon continuous outcomes (e.g. health related quality of life) or a dichotomous outcome (e.g. re-operation, mortality), as calculations using continuous outcomes require smaller sample sizes (often of 80 to 120 patients). Many studies within the orthopaedic trauma literature use continuous outcomes and have sample sizes of approximately 100 patients, which can be sensitive to the results of a few outlier patients [9].

## Conclusions

In conclusion, the data presented from the FLOW study highlight the need for large clinical trials in the field of orthopaedic trauma. Researchers designing a clinical trial need to carefully calculate the sample size to ensure that meaningful results can be achieved to accurately guide clinical practice. Readers of the orthopaedic trauma literature need to critically evaluate the sample size justification as part of their decision to use clinical research to guide the care of their patients.

## Abbreviations

95% CI: 95% confidence interval
COTS: Canadian Orthopaedic Trauma Society
FLOW: Fluid Lavage in Open Fracture Wounds
METRC: Major Extremity Trauma Research Consortium
OTRC: Orthopaedic Trauma Research Consortium
RCT: Randomized controlled trial
REB: Research Ethics Board
RR: Relative risk
SPRINT: Study to prospectively evaluate reamed intramedullary nails in patients with tibial fractures
